# LDLR gene’s promoter region hypermethylation in patients with familial hypercholesterolemia

**DOI:** 10.1038/s41598-023-34639-1

**Published:** 2023-06-07

**Authors:** R. A. Zorzo, V. M. M. Suen, J. E. Santos, W. A. Silva-Jr, V. K. Suazo, A. L. S. C. Honorato, R. D. Santos, C. E. Jannes, A. Pereira, J. E. Krieger, R. D. R. Liberatore-Jr

**Affiliations:** 1 Teaching Institute “Saúde Com Evidência”, Ribeirão Preto, Brazil; 2 Ribeirão Preto Medical School, University of São Paulo University, São Paulo, Brazil; 3 Pediatrics Department, Pediatric Endocrinology, Ribeirão Preto Medical School, São Paulo University, São Paulo, Brazil; 4 Heart Institute (InCor), University of São Paulo Medical School Hospital, São Paulo, Brazil

**Keywords:** Dyslipidaemias, DNA methylation

## Abstract

Familial hypercholesterolemia (FH) is characterized by high low-density lipoprotein cholesterol (LDL-C) levels and a high risk of early coronary heart disease. Structural alterations in the *LDLR*, *APOB*, and *PCSK9* genes were not found in 20–40% of patients diagnosed using the Dutch Lipid Clinic Network (DCLN) criteria. We hypothesized that methylation in canonical genes could explain the origin of the phenotype in these patients. This study included 62 DNA samples from patients with a clinical diagnosis of FH according to the DCLN criteria, who previously tested negative for structural alterations in the canonical genes, and 47 DNA samples from patients with normal blood lipids (control group). All DNA samples were tested for methylation in the CpG islands of the three genes. The prevalence of FH relative to each gene was determined in both groups and the respective prevalence ratios (PRs) were calculated. The methylation analysis of *APOB* and *PCSK9* was negative in both groups, showing no relationship between methylation in these genes and the FH phenotype. As *the LDLR* gene has two CpG islands, we analyzed each island separately. The analysis of *LDLR*-island1 showed PR = 0.982 (CI 0.33–2.95; χ^2^ = 0.001; p = 0.973), also suggesting no relationship between methylation and the FH phenotype. Analysis of *LDLR*-island2 showed a PR of 4.12 (CI 1.43–11.88; χ^2^ = 13,921; p = 0.00019), indicating a possible association between methylation on this island and the FH phenotype.

## Introduction

Familial hypercholesterolemia (FH) affects one in 310 individuals in the general population and is characterized by defects in low-density lipoprotein (LDL) catabolism, resulting in very high levels of LDL cholesterol (LDL-C). FH is associated with the early onset of atherosclerotic cardiovascular disease. Its origin is related, in 60–80% of cases, to structural alterations in three canonical genes (CanGen), *LDLR*, *APOB*, and *PCSK9*, which encode proteins with direct functions in LDL catabolism. However, FH may also be caused by pathogenic mutations in unidentified genes or in several genes, a situation known as Polygenic FH^1,2^.

The clinical diagnosis of FH is usually based on the Dutch Lipid Clinic Network (DCLN) criteria. This method involves a list of criteria leading to a score that eventually defines a diagnosis as “possible” (score 3 to 5), “probable” (score 6 to 8), or “definitive” (score > 8)^2^.

In 60–80% of cases, it is possible to detect variants (changes in sequencing, deletion, or duplication) in at least one CanGen. However, the search for genetic abnormalities is inconclusive in 20–40% of patients with the FH phenotype^3^. One possible explanation is polygenic heritage^4^. Extensive genome-wide association studies (GWASs) of LDL allows the construction of polygenic risk scores (PRSs) for LDL-C in genotyped subjects^5^. Multiple reports have indicated that 20–30% of patients who have clinical FH have a high LDL PRS that may provide the basis of their polygenic hypercholesterolemia^6^.

Another theory is that epigenetic mechanisms such as DNA methylation of CpG islands silence in at least one CanGen, despite its structural integrity.

CpG islands are, on average, 1.000 base pairs of Cytosine and Guanine on promoter region of genes. About 70% of the promoter region of the genes are associated with CpG islands, which makes this mechanism the most common among vertebrates. Their importance lies in the fact that they are sites of initiation of gene transcription, forming part of the sequence of mechanisms that result in chromatin unpacking, creating a possible state for transcription^7^.

Characteristically, CpG islands are not methylated. The unmethylated state is the default that allows the events that culminate in gene transcription to take place^7^.

CpG islands methylation is a mechanism of transcriptional regulation that influences local chromatin spatial structure. This differential configuration of chromatin is an important regulation of gene activity, in fact, the most studied epigenetic mechanism and probably the most frequent. The mechanism is the binding of a methyl radical (CH_3_) to a Cytosine nitrogenous base that precedes a Guanine, resulting in a modification of ionic charges that move away the DNA transcription factors, so that the expression of the gene is not performed, and we say that this gene is “silenced”^8^.

So, to seek for methylation phenomena, one must focus on CpG islands.

*The LDLR* gene, the most common CanGen related to the phenotypic expression of FH, has two CpG islands in the promoter region, which may predispose patients to undergo methylation. *APOB* and *PCSK9* have one CpG island in their respective promoter regions^9^.

Methylation as a cause of the FH phenotype has been investigated by comparing mutation-positive and mutation-negative FH groups, revealing significantly hypomethylated CpG sites in *CPT1A*. No differences were observed in the other genes^10^.

The influence of the FH phenotype in pregnant women on offspring has also been considered. There may be an increased risk among babies exposed to FH during pregnancy, when epigenetic events occur most frequently^11^.

Our hypothesis is that there may be an association between the methylation status of at least one structurally intact CanGen and the FH phenotype. The aim of this study was to test this association.

## Methods

This study investigated the association between the DNA methylation status in structurally intact CanGen s and patients clinically diagnosed with FH according to the DCLN criteria.

Non-probability samples were used for both groups. The inclusion criteria for the study and control groups were the presence and absence, respectively, of a clinical diagnosis of FH according to DCLN scores^12^.

### The analyzed regions of CanGen

The three CanGen were submitted to methylation analysis in each CpG island present in their promoter region.

*LDLR* gene has 44,358 base pairs. Its locus is in chromosome 19 and it is divided into 18 exons and 17 introns. There are two CpG islands in its promoter region, one in intron 1 (with 66 bases) and another in exon 4 (with 29 bases)^9^.

*APOB* gene has 42,645 base pairs. Its locus is in chromosome 2 and codifies two isoforms of lipoprotein B: ApoB-100 and ApoB-48^2^. Its promoter region has one CpG island with 27 bases^9^.

*PCSK9* gene has 25,305 base pairs. Its locus is in chromosome 1. Its promoter region has one CpG island with 85 bases^9^.

**Figure 1:**
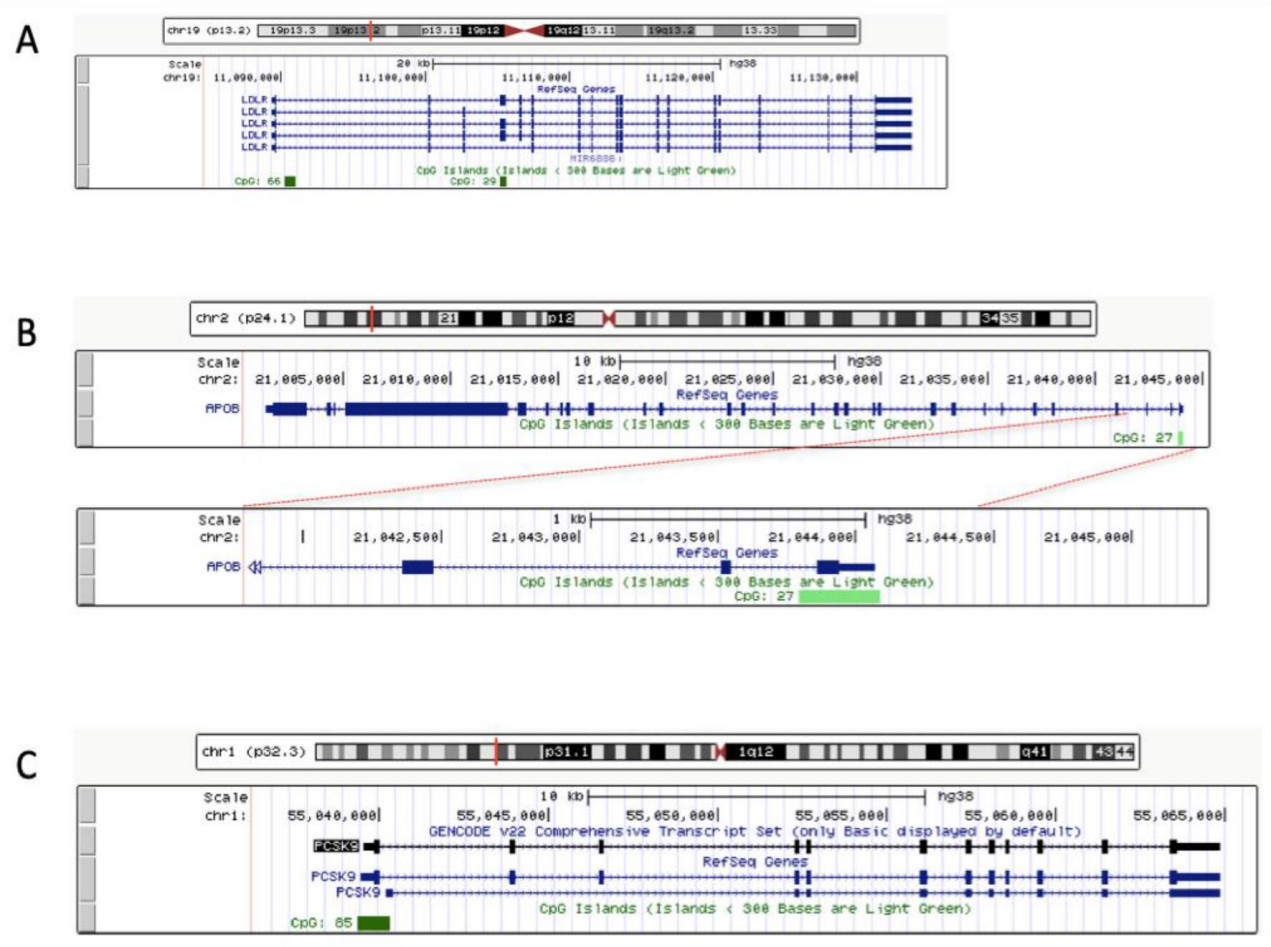
Maps of CanGen’s CpG islands analyzed in this study. (**A**) Presence of two CpG islands in *LDLR* gene (one in intron 1 and another in exon 4). (**B**) One CpG island in *APOB* gene. (**C**) One CpG island in *PCSK9* gene.

These four CpG islands were investigated in this study, which was named as *LDLR*-island1, *LDLR*-island2, *APOB* and *PCSK9*^8^. Maps of each analyzed region are shown in Fig. 1.

### Study group (FH+ group)

The study group included 62 DNA samples from individuals with DCLN scores over 5 and therefore diagnosed as FH “probable” or “definitive.” These samples were obtained from the Hipercol Brasil Project team biorepository. All these samples had previously tested negative for any structural alterations^13^.

Our team received 218 DNA samples from the Hipercol Brasil biorepository. Of these, 156 did not meet the inclusion criteria; 87 DNA samples were from patients who had a DCLN score < 6, 82 had an insufficient amount of genetic material to run the tests, 16 had mutation events, 15 had not been tested for mutation events, 16 were from patients who did not have all the required clinical data, and two were from patients with blood TSH levels > 5.0 mU/L. The 62 remaining DNA samples formed the study group.

All samples were subjected to methylation analysis of the CpG islands of the *APOB* and *LDLR*-island1 genes. Subsequently, one sample was excluded because of insufficient genetic material. The 61 samples remaining were submitted to methylation analysis of the CpG islands *PCSK9* and *LDLR*-island2.

After methylation tests, some DNA samples were excluded from the final analysis: 49 exclusions of *LDLR*-island1 analysis, of which 42 were for anomalous melting curve results and 7 for MT% < 0% or > 120%; 13 exclusions of *APOB* analysis for MT% < 0% or > 120%; and 11 exclusions of *LDLR*-island2 analysis for MT% < 0% or > 120%.

**Figure 2:**
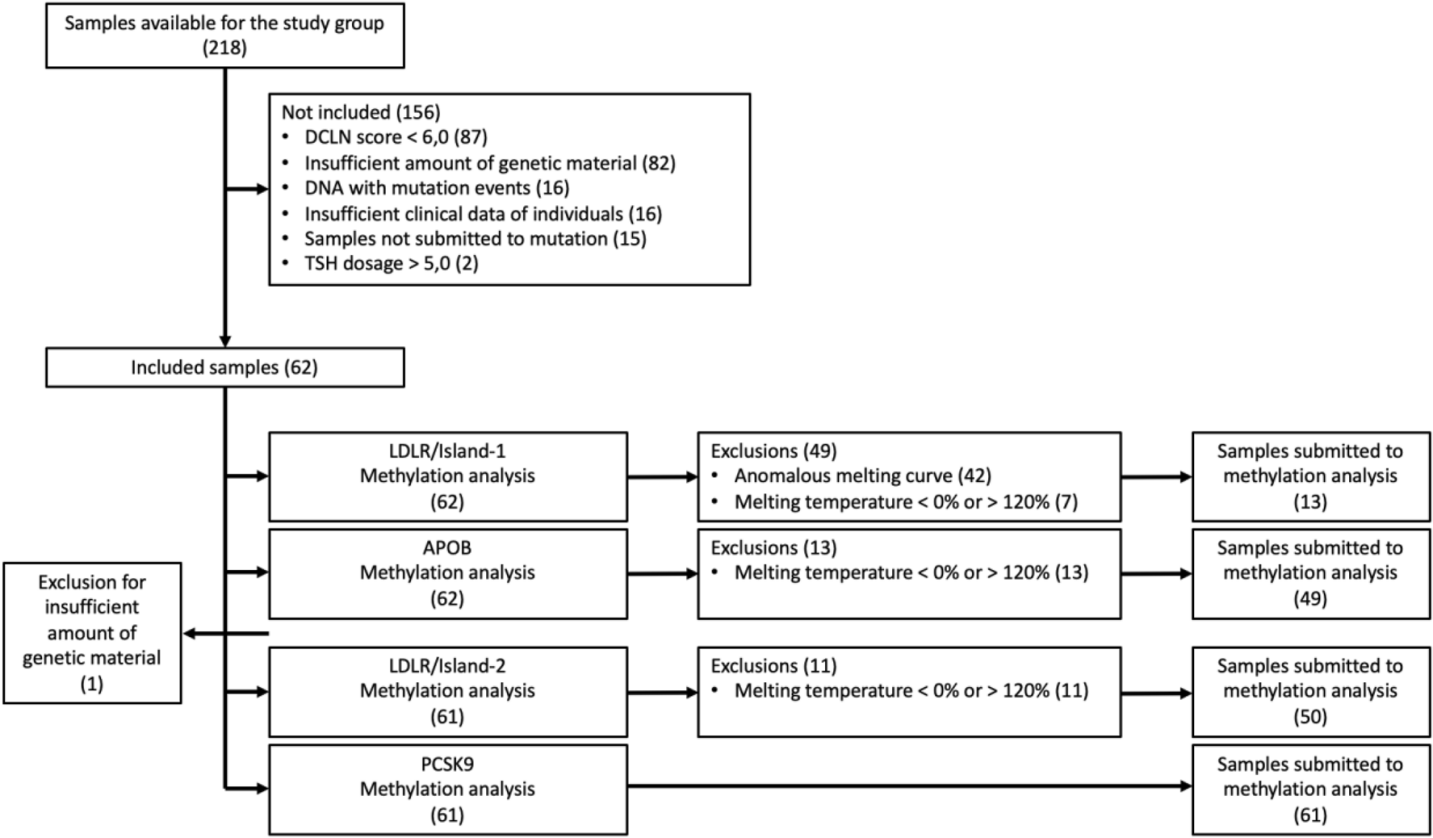
Flowchart of sample inclusion for the FH+ group.

The final study group consisted of 13 samples for *LDLR*-island1, 49 samples for *APOB*, 50 samples for *LDLR*-island2, and 61 samples for *PCSK9*. Figure 2 shows the process for selecting the study group DNA samples.

### Control group (FH− group)

The control group consisted of 47 DNA samples from the Ribeirão Preto School of Medicine biorepository from individuals with at least two previous normal blood LDL-C and triglyceride (TG) levels. We considered this a sufficient condition to assume a negative FH diagnosis. Normal blood levels were LDL-C < 100 mg/dL and TG < 100 mg/dL for individuals between 2 and 20 years of age and LDL-C < 130 mg/dL and TG < 150 mg/dL for individuals over 20 years of age. The mean difference in LDL-C levels between the groups was significant (p < 0.0001). Table 1 presents the details of these populations.

**Table 1:** Clinical and laboratorial characteristics of FH+ and FH− groups.

Characteristics	FH+ Group (n = 62)	FH− Group (n = 47)	p value
Sex			
Male	30 (48.4%)	20 (42.6%)	p = 0.545
Female	32 (51.6%)	27 (57.4%)	
Age, years			
2–20	–	37 (78.7%)	
21–40	11 (17.7%)	10 (21.3%)	
41–60	29 (46.8%)	–	
> 60	22 (35.5%)	–	
*M (σ)*	54.44 (14.85)	14.60 (9.50)	p < 0.001
Ethnicity			
White	36 (58.1%)	38 (80.9%)	p = 0.541
Black/brown	6 (9.7%)	9 (19.1%)	
No ID	20 (32.2%)	–	
BMI (kg/m^2^)			
< 20	2 (3.2%)	NAD	
20–25 (normal)	13 (21.0%)	NAD	
25–30 (overweight)	32 (51.6%)	NAD	
30–35 (class 1 obesity)	12 (19.4%)	NAD	
35–40 (class 2 obesity)	2 (3.2%)	NAD	
> 40 (class 3 obesity)	1 (1.6%)	NAD	
< 100 mg/dL	–	37 (78.7%)	
< 130 mg/dL	–	10 (21.3%)	
LDL-cholesterol			
130–189 mg/dL	–	–	
190–249 mg/dL	22 (35.5%)	–	
250–329 mg/dL	36 (58.1%)	–	
≥ 330 mg/dL	4 (6.4%)	–	
*M (σ)*	295.21 (82.62)	93.57 (18.06)	p < 0.001
Family history of FH	56 (90.3%)	NA	
Clinical history of FH	32 (51.6%)	NA	
Changes in physical examination	4 (6.4%)	NA	

*M* means, *σ* standard deviations. Statistical analyses used: t test for continuous variables and chi-squared test for categorical variables.

*BMI* body mass index, *FH* familial hypercholesterolemia, *LDL* low-density lipoprotein, *NAD* non-available data, *NA* not applicable.

We had 60 DNA samples from patients who had participated in previous clinical trials and were available from the Ribeirão Preto School of Medicine’s biorepository. Of these, 13 were excluded, of which 10 were from patients with blood TG > 150 mg/dL and three from patients with insufficient genetic material. The remaining 47 DNA samples formed the control group and were subjected to methylation analysis of the four CpG islands.

**Figure 3:**
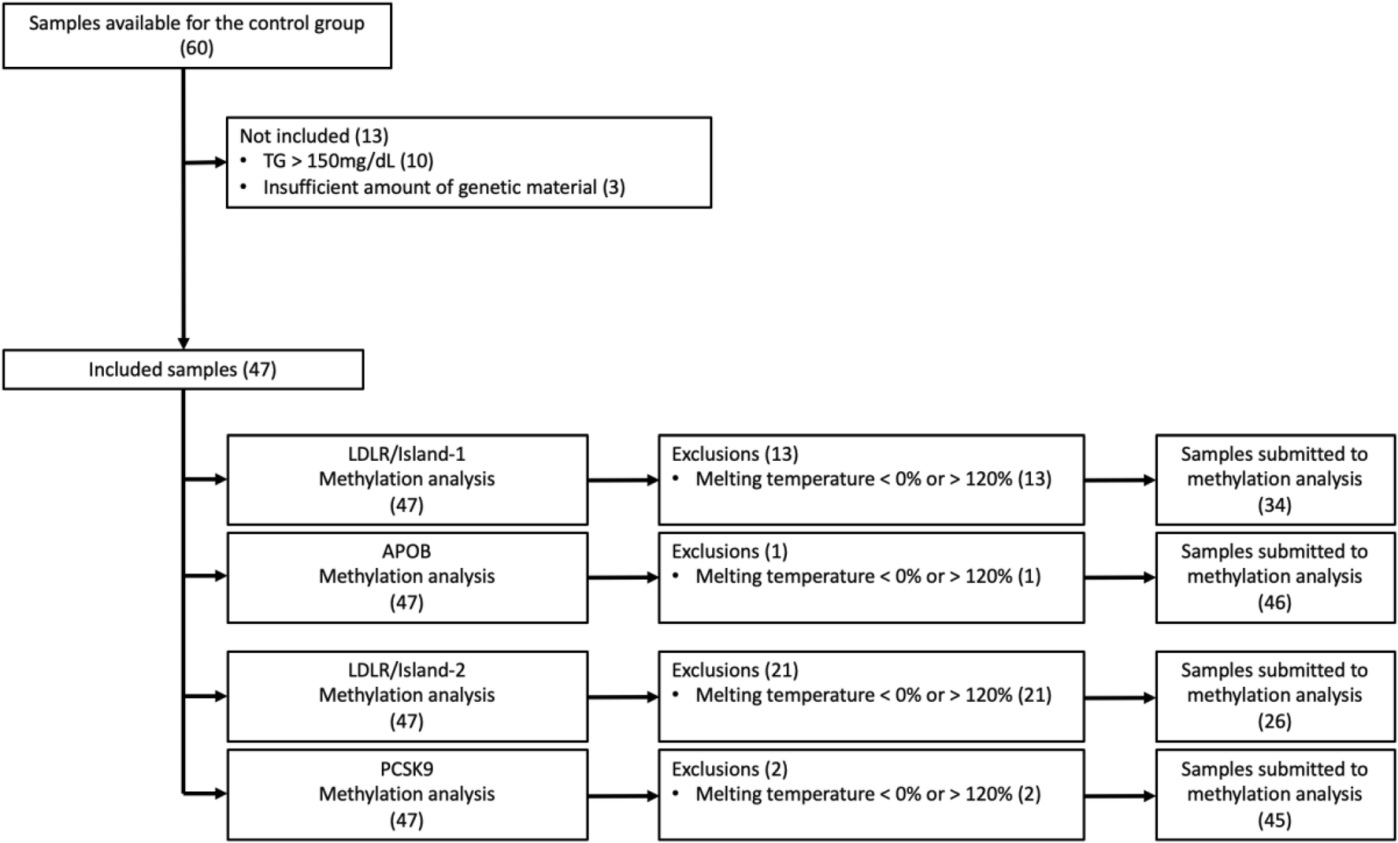
Flowchart of sample inclusion for the FH− group.

After the methylation tests, 13 DNA samples were excluded for *LDLR*-island1, one for *APOB*, 21 for *LDLR*-island2, and two for *PCSK9*. All exclusions were due to MT% < 0% or > 120%. The final control group consisted of 34 samples for *LDLR*-island1, 46 samples for *APOB*, 26 samples for *LDLR*-island2, and 45 samples for *PCSK9*. Figure 3 shows process for selecting the control group DNA samples.

### Analysis of methylation

There are several methods for determining the methylation of DNA samples. Selecting the most appropriate method for answering biological questions appears to be a challenging task. The primary methods in DNA methylation focused on identifying the state of methylation of the examined genes and determining the total amount of 5-methyl cytosine. The study of DNA methylation at a large scale of genomic levels became possible following the use of microarray hybridization technology. Novel sequencing platforms allows the preparation of genomic maps of DNA methylation at the single-open level. The techniques are scrutinized according to their robustness, high throughput capabilities, and cost^14^.

In this study, we proceeded the bisulfite modification method.

The sodium bisulfite reaction modifies the unmethylated Cytosines into Uracil. However, methylated Cytosines do not modify under sodium bisulfite presence, so they stay as Cytosines after reaction^15^.

The importance of this path is the preparation of samples for the next step, which is the melting assay. The melting method is based on the characteristic of DNA double-strains to open into two single strains when submitted to high temperatures. Each nitrogenic base has its own opening temperature, which allows the observer to identify the differences of composition between DNA strains analyzed^16^.

Once unmethylated Cytosines are modified into Uracil, and methylated Cytosines stay stable, melting temperatures will differ between these strains, and so the identification of methylated and unmethylated strains shall be possible^17^.

In this study, the sequence of analysis is described as follow.

All samples from both groups were subjected to modification of genomic DNA with sodium bisulfite^16^. Methylation analysis was conducted using a methylation-sensitive high-resolution melting assay (MeltDoctor HRM Master Mix kit; ThermoFisher®) and polymerase chain reaction (PCR) (7500 Fast Real-Time PCR System; ThermoFisher®). The PCR protocol was to raise the temperature to 95 °C for 10 min, followed by 40 cycles of (1) denaturation at 95 °C for 15 s, (2) annealing at 58 °C for 1 min, and (3) extension at 72 °C for 1 min.

Melting curves were established using the melting temperature (MT) of each sample, with an approximation level of 0.1 °C. MT refers to the system temperature at which 50% of DNA is double-stranded and the other 50% is single-stranded open^18^. The MT of each sample was analyzed using comparisons of 0% and 100% methylated standard MT as parameters^19^.

As the MT is directly related to the methylation status of the sample, it is possible to estimate the percentage of methylated samples by comparing the relative position of the samples’ MT between the respective 0% and 100% standards’ MT^19^.

From MTs of 0% and 100% methylated standards, the methylation percentages of each sample were calculated by proportionality rule^20^, according to the following equation (MT% = sample’s methylation percentage; MTS = sample’s MT; MT0% = 0% methylated standard’s MT; MT100% = 100% methylated standard’s MT):

$$MT\%=\left[MTS-MT0\%\right)/(MT100\%-MT0\%)]$$

We considered samples with MT% ≥ 90% to be methylated. This pattern was considered a positive exposition for the FH phenotype to run the prevalence calculations. Samples with MT% < 0% or > 120% were considered anomalies and excluded from the final analysis.

### Statistical analysis

To compare Groups’ characteristics, we calculated means (M) and standard deviations (σ) of continuous variables, then we run t test with presumably equal variances, and (n1 + n2 − 2) degrees of freedom^21^. The significance level considered was 5% (α = 0.05).

To calculate prevalence, we considered the methylation status of each CpG island separately. Therefore, we determined the prevalence of the FH phenotype and methylation status in four different ways: *APOB*, *PCSK9*, *LDLR*-island1, and *LDLR*-island2.

Statistical analysis included the prevalence ratio (PR) for each CpG island. To test the hypothesis, the respective 95% confidence interval (CI) and chi-squared (χ^2^) test with 1 degree of freedom were calculated^22,23^.

This study was submitted and approved by the Ribeirão Preto Clinics Hospital of São Paulo University’s Ethics Committee (*Comitê de Ética em Pesquisa do Hospital das Clínicas de Ribeirão Preto da Universidade de São Paulo*). Informed consent was waived with accordance of the Ribeirão Preto Clinics Hospital of São Paulo University’s Ethics Committee (*Comitê de Ética em Pesquisa do Hospital das Clínicas de Ribeirão Preto da Universidade de São Paulo*). Original document is available in Supplementary Information.

All methods were performed in accordance with the relevant guidelines and regulations^24^.

## Results

### Statistical analysis of methylation

For *LDLR*-island1, 13 FH + and 34 FH- samples were eligible for determining prevalence. In the study group, three samples were considered methylated, and 10 samples were not methylated. In the control group, eight samples were methylated, and 26 samples were not methylated. The prevalence of disease was 27.3% among those exposed and 27.8% among those not exposed (PR 0.98; CI 0.33–2.95; χ^2^ = 0.001; p = 0.973).

For *APOB*, 49 FH+ and 26 FH− samples were eligible for prevalence determination. None of the samples from either group were methylated.

For *LDLR*-island2, 50 FH+ samples and 46 FH− samples were eligible for determining prevalence. In the study group, 47 samples were considered methylated, and three samples were not methylated. In the control group, 29 samples were methylated and 17 were not methylated. The prevalence of disease 61.8% among those exposed and 15.0% among those not exposed (PR 4.12; CI 1.43–11.88; χ^2^ = 13,921; p = 0.00019).

For *PCSK9*, 61 FH+ and 45 FH− samples were eligible for determining prevalence. None of the samples from either group were methylated.

Table 2 shows the contingency tables.

**Table 2:** Comparisons of methylation patterns and clinical diagnosis for each island analysis.

	FH+	FH−	Total
LDLR/Island-1			
Methylated	3 (27.3%)	8 (72.7%)	11
Not methylated	10 (27.8%)	26 (72.2%)	36
Total	13	34	47
LDLR/Island-2			
Methylated	47 (61.8%)	29 (38.2%)	76
Not methylated	3 (15.0%)	17 (85.0%)	20
Total	50	46	96
APOB			
Methylated	0	0	0
Not methylated	49 (65.3%)	26 (34.7%)	75
Total	49	26	75
PCSK9			
Methylated	0	0	0
Not methylated	61 (57.5%)	45 (42.5%)	106
Total	61	45	106

## Discussion

### Groups formation

Table 1 shows the clinical characteristics of both groups.

Sex distributions were similar on groups: 48.4% of FH+ Group and 42.6% of FH− Group were male.

There was a detectable difference about age distributions on groups. While 17.7% of FH+ Group were between 21 and 40 years-old, 100% of FH− Group were between 2 and 40 years-old. We believe this difference was due to the non-probability origin of the samples. Nevertheless, literature shows that FH+ patients’ LDL-C blood levels should be detected 2–3 times higher than FH- patients even in first years of life^2^. So, we chose the age of 2 years old as the minimum cutoff to enter the study, because from this age the normal levels of LDL-C are known^2^. We considered as inclusion criteria for FH- Group patients who had at least two normal LDL-C blood levels for age (i.e., < 100 mg/dL for age 2–20 years, and < 130 mg/dL for age 21 years or more). For FH+ Group, the minimum LDL-C blood level considered was 190 mg/dL. We did not include patients with LDL-C between 130 and 190 mg/dL in any group. Patients with any family or clinical history of FH, or any change in physical examination that could be an indication of FH were not included on FH- Group, even those who had normal LDL-C levels. We believe that all those cares should minimize the risk of admitting FH+ patients in FH− Group.

The ethnicity criterion also was different between groups, but we believe that this difference had no influence on results, once it is not included in DCLN criteria for FH.

BMI data was only available in FH+ Group. Most patients were in overweight situation (51.6%), while 24.2% was classified as obesity.

The non-probability origin made groups different in several aspects, and they didn’t match in an ideal way to compare biological outcomes. Differences in age, ethnicity and perhaps in BMI could have some influence in LDL-C blood levels. However, we strongly believe that, despite these differences in group characteristics, the methylation status was the principal strength that led to the FH+ phenotype. Some reasonings are: (1) all subjects on FH+ Group had the FH diagnosis based on DCLN criteria, not only on LDL blood levels, and all of them were tested negative to CanGen mutations; (2) LDL differences in groups were different with high statistical significance (p < 0.001); (3) the physiological mechanism under the hypothesis is that the hypermethylation status would silence the expression of LDL receptors, and the study results fit this logic. Furthermore, all possible care to not include some FH+ patient in FH− Group have been taken.

### Analysis of methylation

FH is a common disease associated with early onset of coronary artery disease^2^. The genetic etiology of FH remains unknown in 20–40% of patients with a clinical diagnosis^3^. We postulate that epigenetics might explain some of these cases. DNA methylation in the gene promoter region prevents the opening of the double strand during transcription, resulting in non-expression (or silencing) of this gene^4^.

Among the four CpG islands studied, the methylation pattern of one (*LDLR*-island2) differed between the groups; therefore, the study group (FH+) had a higher proportion of methylation genes, which may demonstrate an association between methylation on this island and the FH phenotype.

A PR of 4.12 indicates that individuals with a methylation pattern higher than 90% had four times the risk of FH when compared to individuals with lower methylation patterns. Silencing of this gene could be a mechanism underlying the FH phenotype^4^.

Differences in the methylation of the other genes were not statistically significant. However, the finding of methylation in specific CpG islands of *LDLR*, which is related to most molecular etiologies of FH, could explain the FH phenotype^2^.

### Limitations

The use of non-probability samples has some limitations. The two groups were not homogeneous because the samples originated from different populations. However, the inclusion criterion established for each group was the presence or absence of a clinical diagnosis of FH. Individuals from the control group had at least two normal LDL-C blood levels, which guaranteed that they did not have FH.2 Working with homogeneous groups and larger samples, it may be possible to observe better results for *LDLR*-island2, *APOB*, and *PCSK9* CpG islands.

Another limitation is that high LDL-C polygenic scores, which may explain up to 20% of the causes of the FH phenotype, were not calculated^3,25^. However, to the best of our knowledge, this is the first study specifically designed to test the association between epigenetic alterations and the FH phenotype.

### Strengths

We found an association between DNA methylation and FH phenotypic expression. Despite the limitations already described, we tested the four CpG islands on the CanGens, which opens the possibility of explaining the origin of the FH phenotype in patients in whom variants in the CanGens were not encountered. Epigenetic mechanisms, particularly CpG methylation, are already well established in the literature.

Considering the role of *LDLR* gene in cholesterol metabolism, we could infer the possible epigenetic role for its methylated status on FH phenotype. Once CpG islands in promoter region have a methylation status, the synthesis of cellular LDL receptors is impaired, resulting in non-recognition of the lipoprotein and an increase in its circulating levels. This could be a novel discovery which would explain the FH+ phonotype in patients with no alterations in DNA structure of CanGen.

It is important to mention the originality of this investigation. There are no other published studies that were designed to test the hypothesis of an epigenetic origin of FH. In view of the exponential increase in publications on epigenetics throughout the 2010s, and the absence of structural alterations in canonical genes in 20 to 40% of patients with positive clinical criteria for FH, it was natural to raise this possibility. The fact that there were no recent related publications caught the attention of the authors, encouraging the design of this study.

The inclusion, non-inclusion and exclusion criteria used were rigorous, to minimize the risk of including subjects with cholesterol alterations not due to FH. Therefore, samples that left doubts about other origins of hypercholesterolemia or that did not have all available data were not included. Among the options of increasing the number of samples, but with partial data, or guaranteeing the quality of the data and increasing the reliability of the FH+ and FH− criteria, even minimizing the final number of samples, the authors chose the second option.

Despite the heterogeneity of the groups, the exploration niche of the hypothesis was preserved by the inclusion and exclusion criteria filters. Even if the subjects who made up the control group had other diseases, the established filters increased the probability that CanGen are free from external influences, as no sample with LDL-C above 100 mg/dL was included.

Finally, there was a positive association finding that ruled out the null hypothesis of the study, with statistical significance, for the established population and criteria.

The authors admit that more studies with larger sample sizes are necessary to better understand the pathogenic mechanisms of FH. However, the novel finding of this study could catch others researcher’s interest to contribute to this point.

## Supplementary Information

Supplementary Information 1.

Supplementary Information 2.

Supplementary Information 3.

Supplementary Information 4.

Supplementary Information 5.

Supplementary Information 6.

## Data Availability

All data generated or analyzed during this study are included in this published article (and its Supplementary Information files).
