# Supplementary material for: LDLR gene’s promoter region hypermethylation in patients with familial hypercholesterolemia
Source: Sci Rep. 2023 Jun 7;13:9241. doi: 10.1038/s41598-023-34639-1 (PMC10247769; doi:10.1038/s41598-023-34639-1)
Supplement: Supplementary file 1 — Supplementary Information 1. [file 41598_2023_34639_MOESM1_ESM.docx]

**Supplementary information #1 – Melting results from LDLR Island 1, FH+ Group**

| **Sample** | **MT(˚C)** | **Met%** | **MET/UNMET** |
| --- | --- | --- | --- |
| 2037 | 72.7 | 100% | MET |
| 2379 | 73.1 | 104% | MET |
| 2441 | 69.5 | 70% | UNMET |
| 2487 | 62.3 | 4% | UNMET |
| 2541 | 69.4 | 69% | UNMET |
| 2873 | 61.9 | 0% | UNMET |
| 2876 | 72.8 | 101% | MET |
| 3001 | 69.2 | 68% | UNMET |
| 3053 | 68.3 | 59% | UNMET |
| 3056 | 67.7 | 54% | UNMET |
| 3064 | 65.3 | 31% | UNMET |
| 3080 | 68.4 | 60% | UNMET |
| 3101 | 69.9 | 74% | UNMET |
