# Supplementary material for: LDLR gene’s promoter region hypermethylation in patients with familial hypercholesterolemia
Source: Sci Rep. 2023 Jun 7;13:9241. doi: 10.1038/s41598-023-34639-1 (PMC10247769; doi:10.1038/s41598-023-34639-1)
Supplement: Supplementary file 2 — Supplementary Information 2. [file 41598_2023_34639_MOESM2_ESM.docx]

**Supplementary information #2 – Melting results from LDLR Island 1, FH- Group**

| **Sample** | **MT(˚C)** | **Met%** | **MET/UNMET** |
| --- | --- | --- | --- |
| 35 | 73.4 | 20% | UNMET |
| 38 | 73.5 | 30% | UNMET |
| 45 | 73.7 | 50% | UNMET |
| 55 | 73.5 | 30% | UNMET |
| 58 | 73.2 | 0% | UNMET |
| 80 | 73.6 | 40% | UNMET |
| 95 | 73.6 | 40% | UNMET |
| 97 | 73.4 | 20% | UNMET |
| 99 | 73.7 | 50% | UNMET |
| 100 | 73.7 | 50% | UNMET |
| 113 | 73.6 | 40% | UNMET |
| 115 | 73.5 | 30% | UNMET |
| 116 | 73.4 | 20% | UNMET |
| 133 | 73.5 | 30% | UNMET |
| 135 | 73.2 | 0% | UNMET |
| 154 | 73.5 | 30% | UNMET |
| 159 | 74.1 | 90% | MET |
| 160 | 73.7 | 50% | UNMET |
| 163 | 74.2 | 100% | MET |
| 169 | 74.2 | 100% | MET |
| 173 | 74.3 | 110% | MET |
| 178 | 73.6 | 40% | UNMET |
| 185 | 73.6 | 40% | UNMET |
| 192 | 73.4 | 20% | UNMET |
| 197 | 73.7 | 50% | UNMET |
| 200 | 73.4 | 20% | UNMET |
| 205 | 73.6 | 40% | UNMET |
| 231 | 74.1 | 90% | MET |
| 233 | 74.2 | 100% | MET |
| 234 | 74.2 | 100% | MET |
| 236 | 73.7 | 50% | UNMET |
| 239 | 74.2 | 100% | MET |
| 251 | 73.4 | 20% | UNMET |
| 268 | 73.4 | 20% | UNMET |
