# Supplementary material for: LDLR gene’s promoter region hypermethylation in patients with familial hypercholesterolemia
Source: Sci Rep. 2023 Jun 7;13:9241. doi: 10.1038/s41598-023-34639-1 (PMC10247769; doi:10.1038/s41598-023-34639-1)
Supplement: Supplementary file 3 — Supplementary Information 3. [file 41598_2023_34639_MOESM3_ESM.docx]

**Supplementary information #3 – Melting results from LDLR Island 2, FH+ Group**

| **Sample** | **MT(˚C)** | **Met%** | **MET/UNMET** |
| --- | --- | --- | --- |
| 2037 | 77.4 | 89% | UNMET |
| 2074 | 77.4 | 89% | UNMET |
| 2379 | 77.9 | 103% | MET |
| 2390 | 77.3 | 86% | UNMET |
| 2441 | 77.9 | 103% | MET |
| 2458 | 77.9 | 103% | MET |
| 2466 | 78.1 | 108% | MET |
| 2479 | 77.9 | 103% | MET |
| 2487 | 77.9 | 103% | MET |
| 2489 | 78.0 | 105% | MET |
| 2503 | 78.4 | 116% | MET |
| 2532 | 77.9 | 103% | MET |
| 2541 | 77.8 | 100% | MET |
| 2574 | 78.1 | 111% | MET |
| 2579 | 78.2 | 114% | MET |
| 2596 | 78.2 | 114% | MET |
| 2599 | 78.2 | 114% | MET |
| 2675 | 78.4 | 119% | MET |
| 2677 | 78.2 | 114% | MET |
| 2685 | 78.1 | 111% | MET |
| 2691 | 78.4 | 119% | MET |
| 2705 | 78.4 | 119% | MET |
| 2732 | 78.2 | 114% | MET |
| 2740 | 78.2 | 114% | MET |
| 2746 | 78.4 | 119% | MET |
| 2790 | 78.1 | 111% | MET |
| 2791 | 77.7 | 100% | MET |
| 2793 | 78.1 | 111% | MET |
| 2794 | 78.2 | 114% | MET |
| 2795 | 78.4 | 119% | MET |
| 2798 | 78.4 | 119% | MET |
| 2806 | 78.3 | 117% | MET |
| 2812 | 78.1 | 111% | MET |
| 2814 | 77.7 | 100% | MET |
| 2823 | 78.2 | 114% | MET |
| 2824 | 78.2 | 114% | MET |
| 2829 | 78.1 | 111% | MET |
| 2851 | 77.5 | 94% | MET |
| 2853 | 77.5 | 94% | MET |
| 2858 | 77.9 | 106% | MET |
| 2866 | 76.6 | 100% | MET |
| 2873 | 76.8 | 106% | MET |
| 2876 | 76.8 | 106% | MET |
| 2882 | 77.1 | 116% | MET |
| 2920 | 77.1 | 116% | MET |
| 3001 | 76.6 | 100% | MET |
| 3016 | 76.8 | 106% | MET |
| 3068 | 77.1 | 116% | MET |
| 3071 | 76.8 | 106% | MET |
| 3080 | 76.8 | 106% | MET |
