# Supplementary material for: LDLR gene’s promoter region hypermethylation in patients with familial hypercholesterolemia
Source: Sci Rep. 2023 Jun 7;13:9241. doi: 10.1038/s41598-023-34639-1 (PMC10247769; doi:10.1038/s41598-023-34639-1)
Supplement: Supplementary file 4 — Supplementary Information 4. [file 41598_2023_34639_MOESM4_ESM.docx]

**Supplementary information #4 – Melting results from LDLR Island 2, FH- Group**

| **Sample** | **MT(˚C)** | **Met%** | **MET/UNMET** |
| --- | --- | --- | --- |
| 14 | 77.9 | 86% | UNMET |
| 23 | 77.9 | 86% | UNMET |
| 25 | 77.9 | 86% | UNMET |
| 32 | 77.9 | 86% | UNMET |
| 35 | 78.3 | 97% | MET |
| 38 | 78.4 | 100% | MET |
| 55 | 78.2 | 94% | MET |
| 58 | 78.0 | 89% | UNMET |
| 80 | 77.9 | 86% | UNMET |
| 83 | 77.9 | 86% | UNMET |
| 86 | 77.8 | 83% | UNMET |
| 92 | 77.9 | 86% | UNMET |
| 94 | 77.5 | 74% | UNMET |
| 95 | 78.4 | 100% | MET |
| 97 | 78.3 | 97% | MET |
| 99 | 78.4 | 100% | MET |
| 100 | 78.4 | 100% | MET |
| 113 | 78.4 | 100% | MET |
| 115 | 78.3 | 97% | MET |
| 116 | 78.3 | 97% | MET |
| 133 | 78.3 | 97% | MET |
| 135 | 77.9 | 86% | UNMET |
| 140 | 77.9 | 86% | UNMET |
| 143 | 77.3 | 69% | UNMET |
| 153 | 77.9 | 86% | UNMET |
| 154 | 78.6 | 97% | MET |
| 159 | 78.4 | 100% | MET |
| 160 | 78.5 | 103% | MET |
| 163 | 78.5 | 103% | MET |
| 169 | 78.5 | 103% | MET |
| 173 | 78.4 | 100% | MET |
| 178 | 78.4 | 100% | MET |
| 185 | 78.3 | 97% | MET |
| 192 | 78.2 | 94% | MET |
| 197 | 77.9 | 86% | UNMET |
| 200 | 77.9 | 86% | UNMET |
| 205 | 78.4 | 100% | MET |
| 231 | 78.4 | 100% | MET |
| 233 | 78.6 | 106% | MET |
| 234 | 78.6 | 106% | MET |
| 236 | 78.6 | 106% | MET |
| 239 | 78.5 | 103% | MET |
| 251 | 78.0 | 92% | MET |
| 260 | 77.8 | 86% | UNMET |
| 262 | 77.2 | 97% | MET |
| 268 | 78.1 | 94% | MET |
