# Supplementary material for: LDLR gene’s promoter region hypermethylation in patients with familial hypercholesterolemia
Source: Sci Rep. 2023 Jun 7;13:9241. doi: 10.1038/s41598-023-34639-1 (PMC10247769; doi:10.1038/s41598-023-34639-1)
Supplement: Supplementary file 5 — Supplementary Information 5. [file 41598_2023_34639_MOESM5_ESM.docx]

**Supplementary information #5 – Contingency tables**

**LDLR Island 1**

|  | FH+ | FH- | Total |
| --- | --- | --- | --- |
| MET | 3 (27.3%) | 8 (72.7%) | 11 |
| UNMET | 10 (27.8%) | 26 (72.2%) | 36 |
| Total | 13 | 34 | 47 |

**LDLR Island 2**

|  | FH+ | FH- | Total |
| --- | --- | --- | --- |
| MET | 47 (61.8%) | 29 (38.2%) | 76 |
| UNMET | 3 (15.0%) | 17 (85.0%) | 20 |
| Total | 50 | 46 | 96 |

**Statistics APOB**

|  | FH+ | FH- | Total |
| --- | --- | --- | --- |
| MET | 0 | 0 | 0 |
| UNMET | 49 (65.3%) | 26 (34.7%) | 75 |
| Total | 49 | 26 | 75 |

**Statistics PCSK9**

|  | FH+ | FH- | Total |
| --- | --- | --- | --- |
| MET | 0 | 0 | 0 |
| UNMET | 61 (57.5%) | 45 (42.5%) | 106 |
| Total | 61 | 45 | 106 |
