# Supplementary material for: LDLR gene’s promoter region hypermethylation in patients with familial hypercholesterolemia
Source: Sci Rep. 2023 Jun 7;13:9241. doi: 10.1038/s41598-023-34639-1 (PMC10247769; doi:10.1038/s41598-023-34639-1)
Supplement: Supplementary file 6 — Supplementary Information 6. [file 41598_2023_34639_MOESM6_ESM.docx]

**Supplementary information #6 – Approbation from Ethics Committee and accordance to waive of Informed consent**
